# Hospitals and Alms-houses in France

**Published:** 1847-04

**Authors:** 


*Hospitals and Alms-houses in France.*—The present number of hospitals or alms-houses in France is 1338, having a revenue of 53,632,992 fr. There are 39 institutions for the education of the deaf, dumb and blind youth of the kingdom, in which at present 1675 pupils are receiving instruction. The number of deaf and dumb persons in France is computed at 20,000 to 25,000, and the number of blind at 12,000 to 15,000. As to the *enfants trouvés*, the number cannot be accurately made out, but 123,394 have been received at the Foundling hospitals, of which there are 144, in a single year. The number of indigent lunatics is 12,286, who are supported by the government at an annual expense of 4,326,138 francs, and by humane and judicious treatment a large number of these unhappy beings are every year restored to reason, and to their families and society.—*Western Journal.*

